# Effects of Single-Course Betamethasone on the Outcomes of Late Preterm Neonates

**DOI:** 10.7759/cureus.46672

**Published:** 2023-10-08

**Authors:** Farinaz Rahimi, Nastaran Safavi Ardabili, Homeira Asgharpoor, Fatemeh Darsareh

**Affiliations:** 1 Mother and Child Welfare Research Center, Hormozgan University of Medical Sciences, Bandar Abbas, IRN; 2 Department of Midwifery, Islamic Azad University, Ardabil, IRN; 3 Mother and Child Welfare Research Center, Hormozgan University of Medical Sciences, Bandar abbas, IRN

**Keywords:** betamethasone, glucocorticosteroid, corticosteroid, neonatal outcome, preterm birth

## Abstract

Background

Prenatal glucocorticoids are commonly used in pregnancies before 34 weeks of gestation in women at risk of preterm delivery; however, the effects of these drugs on late preterm infants (those born after the 34th week of pregnancy) are controversial. As a result, we aimed to investigate the effect of a single course of betamethasone (two doses of betamethasone every 24 hours) on the neonatal outcome of late preterm births.

Methods

We retrospectively assessed all spontaneous late preterm births (34-36+6 weeks of gestation) at a tertiary hospital in Iran over a period of two and a half years. Exclusion criteria included multiple pregnancies, induced labor, and fetal malformations identified in sonograms. Neonatal outcome measures encompassed first and five-minute Apgar scores, respiratory distress syndrome, neonatal death, birth asphyxia, and the need for positive pressure ventilation, continuous positive airway pressure, tracheal intubation, and surfactant. Baseline characteristics, such as maternal age, parity, fetal gender, and high-risk pregnancy, were considered confounding variables. High-risk pregnancies were defined as any cases involving prolonged rupture of membranes or maternal comorbidities such as severe anemia, preeclampsia, diabetes, or COVID-19.

Results

During the study period, there were 830 spontaneous preterm births at our center. Of these, only 195 (23.5%) received complete doses of betamethasone. Low birth weight was more common in mothers who did not receive betamethasone compared to those who did (63.6% vs. 41.2%). The mean gestational age was lower in mothers who received betamethasone than in those who did not. Respiratory distress syndrome was more common in mothers who received betamethasone (P<0.001, RR 2.11, 95% CI (0.98-4.18)). However, after adjusting for confounding factors, such as gestational age and birth weight, betamethasone did not increase the risk of respiratory distress syndrome. Other adverse neonatal outcomes did not differ significantly.

Conclusions

There were no differences in neonatal outcomes between those who received betamethasone and those who did not.

## Introduction

Antenatal glucocorticosteroids used in pregnancies at risk for preterm birth reduce the risk of preterm newborn mortality and morbidity [1,2]. Given these advantages, numerous clinical guidelines recommend antenatal glucocorticosteroids in pregnancies at risk of preterm birth. A single course of betamethasone is recommended by the American Congress of Obstetrics and Gynecology for all pregnant mothers between 24 weeks and 36+6 weeks of gestation who might be at risk of preterm delivery within seven days and have not previously received prenatal glucocorticosteroids [3]. According to the latest study, prenatal glucocorticosteroid administration from 34 to 36+6 weeks gestation also reduces the risk of neonatal respiratory distress but increases the risk of neonatal hypoglycemia. The long-term effects of antenatal glucocorticosteroid administration between weeks 34 and 36+6 are unknown [2]. On the other hand, a recently published study indicated that using betamethasone in the late preterm period (after 34 weeks of gestation) has no benefits on the promotion of fetal lung maturity or preventing the neonatal negative adverse events and may even increase the risk of respiratory distress syndrome and the need for respiratory support [4]. Prenatal glucocorticoids are commonly used in pregnancies before 34 weeks of gestation in mothers at risk of preterm birth; however, the effects of these drugs on late preterm infants (those born after the 34th week of pregnancy) are controversial. As a result, we aimed to investigate the effect of a single course of betamethasone on the neonatal outcome of late preterm birth.

## Materials and methods

The Ethics and Research Committee of the Hormozgan University of Medical Sciences reviewed and approved the study (approval no. HUMS.REC.1402.115). Due to the study's retrospective nature, the informed consent was waived. Statistical analysis was performed with patient anonymity following ethics committee regulations. We conducted a retrospective assessment of all spontaneous late preterm births (34-36+6 weeks of gestation) that occurred over two and a half years at a tertiary hospital in Bandar Abbas, Iran. Exclusion criteria included multiple pregnancies, labor-induced due to maternal or neonatal complications, fetal malformations identified in sonograms, and incomplete doses of betamethasone. Trained data collectors used electronic patient records to extract data from the 'Iranian Maternal and Neonatal Network (IMaN Net),' a credible national system. Neonatal outcome measures included Apgar scores at one and five minutes, respiratory distress syndrome, neonatal death, birth asphyxia, and the need for positive pressure ventilation (PPV), continuous positive airway pressure (CPAP), tracheal intubation, and surfactant. We considered baseline characteristics such as maternal age, parity, fetal gender, and high-risk pregnancy as confounding variables. High-risk pregnancies were defined as those involving prolonged rupture of membranes or maternal comorbidities, such as severe anemia, preeclampsia, diabetes, or COVID-19. Glucocorticosteroid administration guidelines and types have remained largely unchanged since their discovery [5]. Current international recommendations advise initiating treatment with 12 mg of betamethasone every 24 hours for two doses or four doses of 6 mg of dexamethasone every 12 hours for any gestation at risk of preterm delivery [6-9]. In our study, we included mothers who received a regimen of two doses of betamethasone, injected intramuscularly every 24 hours. Those with an incomplete dose of betamethasone were excluded from the analysis. We used SPSS, version 19 (IBM Corp, Armonk, NY), for data analysis. Categorical variables are presented as numbers and frequencies (%). Chi-square tests were used to compare differences between mothers who received betamethasone and those who did. We compared the continuous variables using the t-test or Mann-Whitney U test if the data did not appear to have normal distribution or when the assumption of equal variances was violated across the study groups. The effect of exposure to betamethasone was expressed using relative risk (RR). A 95% CI and P < 0.05 were considered statistically significant; all statistical tests were two-tailed.

## Results

From January 2020 to January 2022, 830 spontaneous preterm births occurred at our center. Of these, only 195 (23.5%) mothers received a complete dose of betamethasone. Table 1 compares the maternal characteristics of the mothers receiving and not receiving betamethasone. As shown, neonatal birth weight was the factor that differed significantly. Low birth weight was more common in mothers who did not receive betamethasone than those who received betamethasone (63.6% vs. 41.2%). The mean gestational age in mothers who received betamethasone was lower than those who did not.

**Table 1:** Maternal characteristics in the two groups of mothers receiving and not receiving betamethasone.

Characteristics	Without Betamethasone (n=635)	With Betamethasone (n=195)	P-value
Age (%)			0.444
13-19 Year	8 (1.3)	4 (2.1)	
20-34 Year	494 (77.8)	148 (75.9)	
35-40 Year	106 (16.7)	30 (15.4)	
Above 40 Year	27 (4.3)	13 (6.7)	
Highrisk pregnancy (%)			0.121
Yes	204 (32.1)	59 (30.2)	
No	431 (67.9)	136 (69.8)	
Parity			0.087
Primiparous	168 (26.5)	62 (31.8)	
Multiparous	465 (73.5)	133 (68.2)	
Neonatal weight (%)			
1500-2500 grams	262 (41.2)	124 (63.6)	<0.001
More than 2500 grams	373 (58.8)	71 (36.4)	
Fetal gender (%)			0.085
Female	308 (48.5)	83 (42.6)	
Male	327 (51.5)	112 (57.4)	
Gestational age (Mean±SD)	35.56±0.65	34.66±0.72	<0.001

Neonatal outcomes of mothers who received betamethasone and those who did not are illustrated in Table 2. Respiratory distress syndrome was more common in mothers who received betamethasone (P<0.001, RR 2.11 95% CI (0.98-4.18)). However, after adjusting for confounding factors (gestational age and birth weight), betamethasone did not increase the risk of respiratory distress syndrome. Other adverse neonatal outcomes did not differ significantly. 

**Table 2:** Neonatal outcomes in the two groups of mothers receiving and not receiving betamethasone. Data are presented as n (%) or Mean±SD RR: Relative risk; ARR: Adjusted relative risk; RDS: Respiratory distress syndrome; PPV: Positive pressure ventilation; CPAP: Continuous positive airway pressure.

Outcome	Without Betamethasone (n=635)	With Betamethasone (n=195)	P-value	RR 95% CI	ARR 95% CI	P-value
RDS	245 (38.6)	135 (69.2)	<0.001	2.11 (0.98-4.18)	0.98 (0.21-1.35)	0.167
Neonatal death	6 (0.9)	0	0.781	Not Assigned	Not Assigned	Not Assigned
Tracheal intubation	9 (1.4)	2 (1.0)	0.879	Not Assigned	Not Assigned	Not Assigned
Asphyxia	15 (2.4)	8 (4.1)	0.197	Not Assigned	Not Assigned	Not Assigned
First minute Apgar	8.29±1.92	8.36±1.14	0.525	Not Assigned	Not Assigned	Not Assigned
Fifth minute Apgar	9.28±2.03	9.43±0.85	0.141	Not Assigned	Not Assigned	Not Assigned
Need for PPV	23 (3.6)	8 (4.1)	0.763	Not Assigned	Not Assigned	Not Assigned
Need for CPAP	98 (15.4)	38 (19.5)	0.098	Not Assigned	Not Assigned	Not Assigned
Need for Surfactant	6 (0.9)	4 (2.0)	0.132	Not Assigned	Not Assigned	Not Assigned

## Discussion

The administration of antenatal glucocorticosteroids (betamethasone or dexamethasone) to pregnant mothers before preterm delivery is widely regarded as one of the most significant advances in maternity care. Glucocorticosteroids may promote lung microbial growth by increasing alveolar type II pneumocyte cell maturation and surfactant production, improving antioxidant activity, and decreasing pro-inflammatory cytokine production [10]. A substantial body of evidence from multiple randomized controlled trials supports using glucocorticosteroids such as betamethasone or dexamethasone before early preterm birth (24-34 weeks of pregnancy) to decrease neonatal mortality, neonatal respiratory disease, and other adverse events of newborns [11], with little or no evidence of long-term harm [12]. As a result, clinical practice guidelines recommend using them as a standard, whenever possible, for all mothers at a continued risk of early preterm delivery [3]. However, the story differs for late preterm (after 34 weeks of pregnancy). As observed in our study, the rate of adverse neonatal effects in mothers who received betamethasone was comparable to those who did not. There was no beneficial effect of prenatal administration of betamethasone for late preterm delivery. However, unlike other studies, no harm was observed. Some studies found more significant adverse neonatal outcomes in mothers who received glucocorticosteroids than those who did not [4,13-15]. For example, in 1972, Liggins GC and Howie RN designed and conducted the first randomized controlled trial of antenatal glucocorticosteroid administration to improve fetal pulmonary maturation. According to them, betamethasone administration reduced the rate of respiratory distress syndrome in the intervention group compared to the control group. When they performed a subgroup analysis on neonates between 32 and 37 weeks gestation, they discovered a lower rate of respiratory distress syndrome, dropping from 6.9% in the control group to 4.7% in the intervention group [13]. Similar to this report, another observational study on 5,924 participants found that antenatal glucocorticosteroid exposure does not affect respiratory outcomes in those with a subsequent late preterm delivery [14]. A retrospective cohort study conducted by Arimi Y et al. examined 100 consecutive newborns, aged 34 to 36 weeks of gestation, who received a single course of betamethasone prior to birth and compared them to a control group of 100 newborns of the same gestational age who did not receive betamethasone. Their findings indicated that the use of a single course of betamethasone during the late preterm period did not positively impact the promotion of fetal lung maturity. Furthermore, it did not provide a beneficial effect in preventing adverse events in newborns and may even increase the risk of respiratory distress syndrome [4].

In contrast, others found that the rate of respiratory problems in the group receiving prenatal glucocorticosteroids was significantly lower than in the control group and that glucocorticosteroids cause lung maturation even during late preterm [3,15]. Lastly, a single large trial conducted in the United States showed a positive effect of glucocorticosteroids in preventing respiratory syndrome disease in late preterm pregnancies [3]. This finding prompted the American College of Obstetricians and Gynecologists (ACOG) Committee to adopt recommendations for practice change. Many expert commentaries and reviews have expressed reservations about the rapid adoption of practice change, especially when realistic concerns about the risk/benefit ratio remain unresolved. As a result, additional research is advised in order to reach a more accurate conclusion. As with many studies, our study is not free of limitations. The fact that we do not know the gestational age at which the betamethasone was administered, and thus the time interval between betamethasone administration and delivery, is a limitation of our study. This is a significant confounder because the effects of betamethasone administration are thought to be transient. Another limitation is that we did not investigate the long-term effects. The small sample size is another limitation.

## Conclusions

In contrast to studies that supported betamethasone administration, emphasizing its efficacy in mitigating neonatal complications in late preterm births, and opposing those reporting a higher incidence of adverse effects in infants following betamethasone use, our findings revealed no differential in neonatal outcomes between subjects who received betamethasone and those who did not. Further studies with a larger sample size may lead us to a better conclusion. We recommend randomized clinical trials that explore the impact of varying courses of glucocorticosteroids on late preterm births.
